# Novel indole-bearing combretastatin analogues as tubulin polymerization inhibitors

**DOI:** 10.1186/2191-2858-3-3

**Published:** 2013-03-03

**Authors:** Sunil Kumar, Samir Mehndiratta, Kunal Nepali, Manish K Gupta, Surrinder Koul, Parduman R Sharma, Ajit K Saxena, Kanaya L Dhar

**Affiliations:** 1 Laboratory for Drug Design and Synthesis, Department of Pharmaceutical Chemistry, Indo-Soviet Friendship (ISF) College of Pharmacy, Moga, Punjab 142 001, India; 2 Division of Bio-Organic Chemistry and Pharmacology, Indian Institute of Integrative Medicine (IIIM), CSIR, Jammu Tawi 180 001, India

**Keywords:** Colchicine binding site, Indole-based combretastatin, Molecular docking, Tubulin inhibitors

## Abstract

**Background:**

The combretastatins are a class of natural stilbenoids. These molecules generally share three common structural features: a trimethoxy "A"-ring, a "B"-ring containing substituent often at C3′ and C4′, and an ethene bridge between the two rings, which provides necessary structural rigidity. Members of the combretastatin family possess varying ability to cause vascular disruption in tumors. Combretastatin binds to the colchicine binding site of *β*-subunit of tubulin. Despite having a similar name, combretastatin is unrelated to statins, a family of cholesterol-lowering drugs.

**Results:**

New combretastatin 2-(1-acetyl-1*H*-indole-3-yl)-3-(phenyl) propenoic analogues (**2a** to **2y**), bearing indole moiety at the place of ring A of combretastatin (CA4), were synthesized and evaluated for anticancer activity against various cancer cell lines such as THP-1 (leukemia), A-549 (lung), IGROV-1 (ovary), HEP-2 (liver), MCF-7 (breast), and DU-145 (prostate). Compound **2d** showed anti-cancer activity against THP-1 and MCF-7 with IC_50_ 0.80 and 0.37 μM, respectively, and **2y** showed against MCF-7 with IC_50_ 3.60 μM comparable to paclitaxel.

**Conclusions:**

The target compounds bind to the colchicine binding site which is situated at *α* and *β* interface of tubulin and prevent polymerization as it was confirmed by immunofluorescence technique. The molecular docking further confirmed the binding of the potent compound **2d** to the colchicine binding site at *α* and *β* interface of tubulin.

## Background

**Figure 1:**
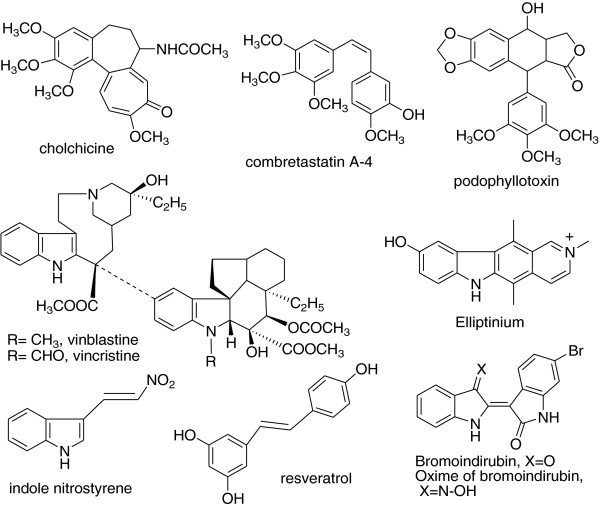
Some reported tubulin inhibitors.

Tubulin is a useful biochemical target for various clinically used anticancer drugs like paclitaxel, vincristine, and vinblastine (Figure 1) [1]. It plays an important role in the formation of the mitotic spindle which provides the structural framework for the physical segregation of the chromosomes during the mitosis [2]. Microtubules, made from tubulin are highly dynamic cytoskeleton elements in eukaryotic cells and play a critical role in various processes like mitosis, cell shape, intracellular organelle, transport and cell-cell interactions, and signal transduction [3,4]. They have highly polar structure and favor growth at the plus (+) end and shrinkage at the minus (−) end. This dynamic equilibrium can be affected by treatment with some agents like phenstatin, chalcone, lignan, colchicine, and podophyllotoxin ultimately prevent mitosis, thus used for cancer treatment [5,6]. Drugs which target tubulin interact with mitotic spindle and act by binding to the following binding sites: (1) colchicine binding site, (2) vinca alkaloid binding site, (3) rhizoxin/maytansine binding site, (4) tubulin sulfhydryl binding site, and (5) unknown binding sites [1]. Taxol binds to the *β*-tubulin of colchicine binding site and stabilizes microtubule against depolymerization. Colchicine binds to the *α*-tubulin of colchicine binding site and blocks the polymerization of microtubule [7]. Combretastatins binds to *β*-tubulin of colchicine binding site, strongly inhibits tubulin polymerization [8-10], and disrupts the normal mitotic spindle function [11]. It was first isolated from the bark of African willow tree *Combretum caffrum*[12]. Various analogues have been synthesized and showed very good anticancer activity by inhibiting tubulin polymerization [13]. Earlier synthesized compounds were having two rings in *cis*-configuration, but Pettit et al. have synthesized resveratrol type of compounds, *β*-nitrostyrenes, in which two rings are in *trans*-configuration and have marked antitumor activity. They also have synthesized E-indole nitrostyrene which showed marked anticancer activity [14]. Anticancer agents vincristine and vinblastine derived from *Catheranthus roseus* have been useful in cancer treatment, and this effect is due to the interaction of these drugs with tubulin [8,15]. It was thought worthwhile to incorporate another ring system having indole nucleus to rationally design combretastatin-like compounds replacing one ring with indole moiety because vincristine, vinblastine, elliptinium [16], and bromoindirubin contain indole moiety and have good tubulin polymerization inhibitory property [17]. Resistance remains a significant problem in the treatment of cancer with taxol like many chemotherapeutic agents, and it is caused by alterations in microtubule dynamics [18]. Our aim was to synthesize tubulin inhibitors having indole ring as basic moiety.

## Methods

**Scheme 1:**
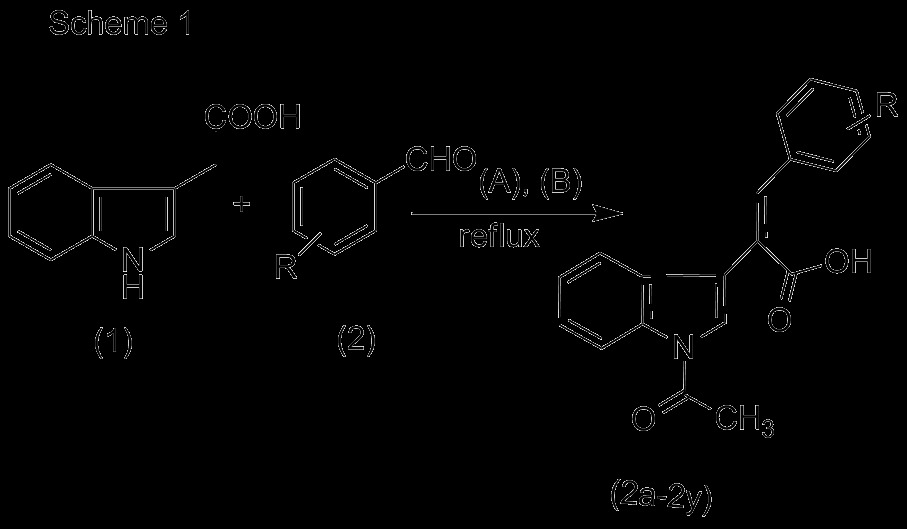
**Reagents and conditions. **(**A**) Triethylamine; (**B**) acetic anhydride, refluxed for 6 to 10 h.

Combretastatin 2-(1-acetyl-1*H*-indole-3-yl)-3-(phenyl) propenoic analogues (**2a** to **2y**) were synthesized by condensation of indole-3-acetic acid with different substituted aldehydes using triethyl amine and acetic anhydride (shown in Scheme 1). For anticancer activity, seven cancer cell lines like lung (A-549), ovary (IGROV-1), prostate (DU-145), liver (HEP-2), leukemia (THP-1), and breast (MCF-7) were used.

### Materials and methods of immunofluorescence confocal microscopy

THP1 cells (8 × 10 [4] cells/well) were seeded onto 18-mm square coverslips in six well plates. Cells were allowed to adhere for 24 h before dosing with required concentrations of **2d**. Paclitaxel 1 μM was used as a positive control. After the treatment period, cells were fixed in 4% paraformaldehyde for 10 min at room temperature and permeabilized using 0.5% Triton-X (Sigma-Aldrich, MO, USA) in PBS for 5 min. The cells were blocked with 10% goat serum for 20 min at room temperature. Microtubules were detected with a monoclonal *α*-tubulin antibody (Sigma Corporation, Cream Ridge, USA) diluted 1:100 in 0.1% Triton X-100 in PBS for 1 hr 37°C and Alexa Fluor 488 conjugated secondary antibody (Invitrogen, Carlsbad, USA) diluted 1:1,000 in PBS for 1 hr at room temperature. Cells were then washed three times in PBS and stained with DAPI diluted 1:1,000 in PBS [5]. The coverslips were mounted over glass slides, and the cells were imaged by confocal microscopy using an Olympus Fluoview FV1000 laser scanning microscope (Olympus Inc., Center Valley, USA).

### Material and methods for molecular docking

The coordinates of tubulin were obtained from protein data bank [PDB:1SA0] [19,20]. The structure of **2d** was drawn in ChemDraw [21] and subjected to energy minimization in the MOPAC module, using the AM1 procedure and implemented in the CS Chem3D Ultra (Cambridge Soft Corporation, Cambridge, USA). The **2d** was docked in to the colchicine binding site of tubulin using the GOLD 5.0.1 software [22]. GOLD performs genetic algorithm-based ligand docking to optimize the conformation of the ligand at the receptor binding site. It utilizes Gold score fitness function to evaluate the various conformations of the ligand at the binding site and comprises four components: protein-ligand hydrogen bond energy, protein-ligand van der Waals (vdw) energy, ligand internal vdw energy, and ligand torsional strain energy. The compound was docked ten times, and each pose was ranked according to its Gold score fitness function. The conformation with the highest score was selected for discussion.

## Results and discussion

### Chemistry

**Figure 2:**
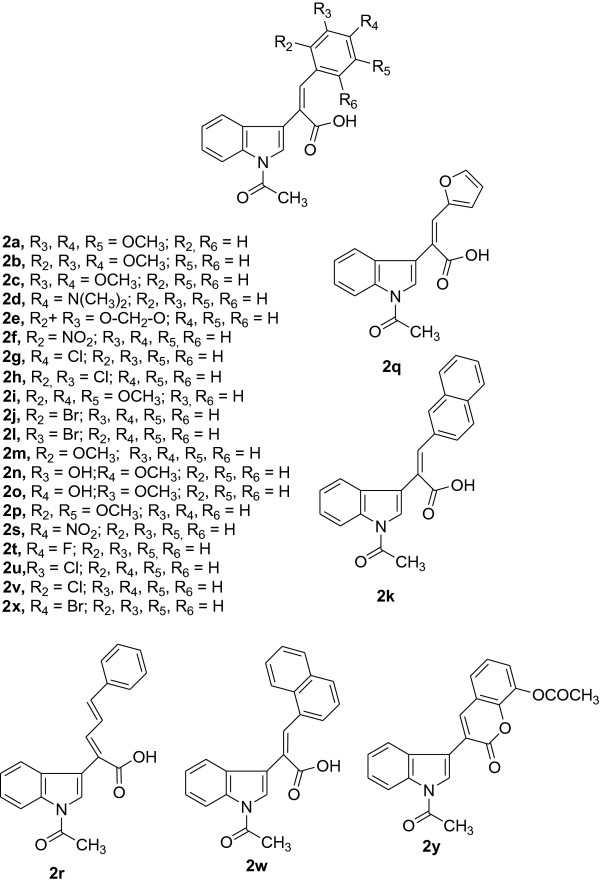
Chemical structures of the synthesized compounds.

**Figure 3:**
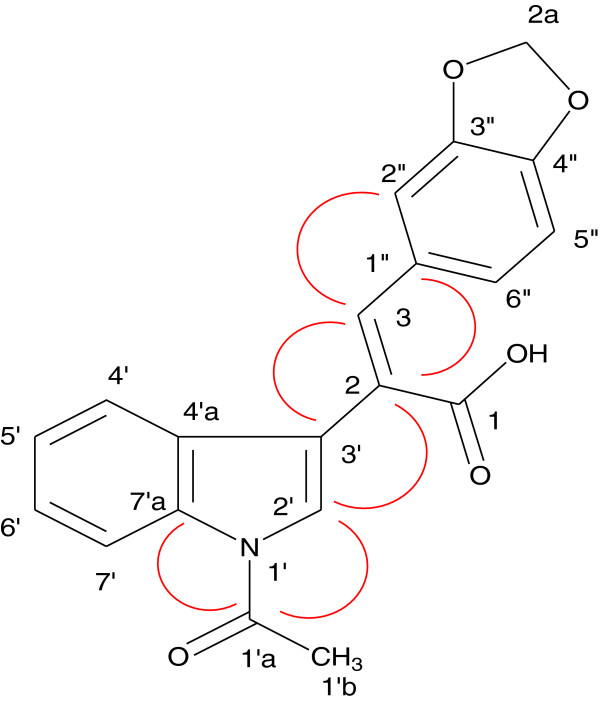
**Selected HMBCs of 2e.** Red ring indicates the correlation of two carbons or hydrogens which are near to each other.

**Figure 4:**
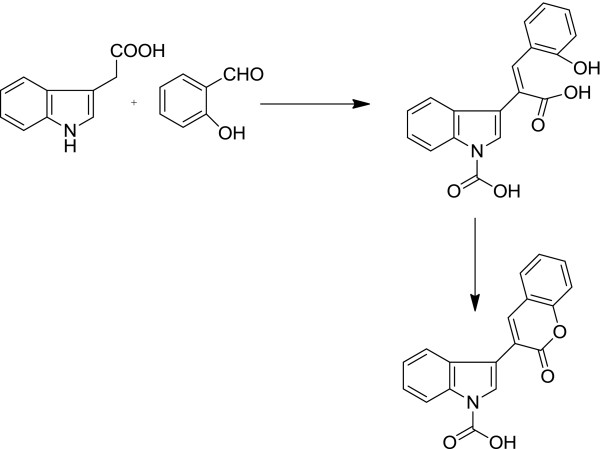
Lactonization of combretastatin analogue.

Twenty-five combretastatin analogues (**2a** to **2y**) were synthesized by condensation of indole-3-acetic acid (1) with different substituted aldehydes (2) using triethyl amine and acetic anhydride as reported previously (shown in Scheme 1) [23]. The reaction was monitored by TLC. The compounds were purified (yield 48% to 63%) by column chromatography using silica gel mesh size of 60 to 120. All the synthesized compounds (Figure 2) were characterized by spectroscopic techniques like infrared spectrometry (IR), mass spectrometry (MS), and nuclear magnetic resonance spectroscopy (NMR) (1H NMR, 13C NMR, heteronuclear multiple bond correlation (HMBC) NMR (Figure 3 and Table 1), and heteronuclear single quantum coherence (HSQC). 1H NMR indicates that H-7′ is highly deshielded as it appears to lie in the deshielding cone of N-acetyl group. H-3 is deshielded because of its nearby carbonyl of N-acetyl. H-3 is also deshielded because of its *β* position to carbonyl, but it is less deshielded than H-7′ because it does not come under the influence of magnetic anisotropy of carbonyl. No change in the integration of the H-3 was observed when H-2′ was doubly irradiated. These results indicated that H-2′ and H-3 are not in the close proximity. This is possible only when two rings are *trans* to each other. If two rings would have been in *cis* configuration, double irradiation of H-2′ would have resulted in signal enhancement of H-3 due to nuclear Overhauser effect (nOe). It was further confirmed that when a compound with hydroxyl located at 2″ was synthesized, the free OH (at 2″) is used in the formation of lactone because the geometry is such where OH and C = O are close in space and prefer to form lactone. H-3 gets more deshielded than H-7′ because it comes in the deshielding cone in the plane of benzene ring as well as the deshielding cone of carbonyl. IR value of carbonyl shifted to 1,763 from 1,710 because of lactone formation. Further Pschorr reaction [24] is fully supported in the condensation of phenyl acetic acid with aldehyde, resulting into combretastatin with *cis* configuration. When 2-hdroxybenzaldehyde is used for the condensation with phenyl acetic acid, no lactone formation occurred (Figure 4). From the above observations, it was thus concluded that synthesized compounds are having two rings in *trans*-configuration.

**Table 1:** **Three-bond**[1]**H-**[13]**C coupling (HMBC) in 2e**

**Serial number**	**H**	**C**
1	H-3	C-3’, C-2″
2	H-2’	C-1’a, C-2
3	H-4’	C-3’
4	H-7’	C-1’a
5	H-2″	C-3
6	H-6’’	C-3, C-1

### Anticancer activity

All the synthesized compounds were evaluated for anticancer activity (Table 2) against lung (A-549), ovary (IGROV-1), prostate (DU-145), liver (HEP-2), leukemia (THP-1), and breast (MCF-7) cancer cell lines. Compounds **2j**, **2 k**, and **2r** showed good anticancer activity against THP-1, DU-145, and MCF-7 cell lines. However, compounds **2d** (IC_50_ = 0.80 and 0.37 μM) and **2y** (IC_50_ = 3.6 μM) showed marked anticancer activity.

**Table 2:** Anticancer activity of synthesized compounds

**Compound code**			**IC**_**50 **_**of cell line types (tissue/cell)**			
	**THP-1**	**A-549**	**MCF-7**	**IGROV-1**	**HEP-2**	**DU-145**
	**(WBC)**	**(lung)**	**(breast)**	**(ovary)**	**(liver)**	**(prostrate)**
**2a**	>100	>100	86.9	>100	>100	>100
**2b**	>100	>100	>100	>100	-	-
**2c**	89.2	71.0	>100	>100	-	-
**2d**	*0*.*80*	>100	*0*.*37*	38.0	63.4	40.4
**2e**	73.0	>100	>100	>100	-	-
**2f**	81.3	>100	>100	>100	>100	>100
**2 g**	70.6	>100	>100	-	89	>100
**2 h**	>100	>100	>100	-	>100	-
**2i**	-	-	>100	-	-	-
**2j**	>100	>100	76.1	-	-	16.4
**2 k**	>100	>100	50.0	-	-	43.9
**2 l**	86.0	>100	76.4	>100	>100	>100
**2 m**	67.0	75.7	>100	>100	>100	>100
**2n**	>100	>100	>100	>100	-	-
**2o**	>100	>100	>100	>100	-	-
**2p**	>100	>100	>100	>100	-	-
**2q**	68.0	>100	79.0	>100	>100	>100
**2r**	40.8	38.7	51.9	>100	>100	>100
**2 s**	>100	90.3	>100	>100	>100	>100
**2 t**	-	>100	>100	-	>100	>100
**2u**	>100	>100	>100	>100	>100	>100
**2v**	>100	>100	95.2	>100	-	-
**2w**	>100	>100	51.8	>100	-	-
**2x**	92.0	>100	>100	>100	>100	>100
**2y**	81.8	49.8	*3*.*6*	>100	85.3	-
Adriamycin	*0*.*12*	*0*.*11*	*0*.*16*	*0*.*19*	-	-
Mitomycin	*0*.*23*	*0*.*19*	*0*.*27*	*0*.*25*	*0*.*31*	-
Paclitaxel	*0*.*15*	-	-	*0*.*15*	*0*.*14*	-
5-fluorouracil	*9*.*7*	*8*.*6*	*9*.*9*	*8*.*3*	*9*.*7*	*9*.*1*

The italicized values indicate significant activity.

Synthesized compounds, in spite of having basic indole moiety, bind to colchicine binding site instead of vinblastine binding site because vincristine and vinblastine molecules are very large molecules, and thus, their binding sites have enlarged cavity as the binding site, while the present synthesized compounds, being much smaller in overall dimensions, appear to roll around in the vinblastine site. Further, this was confirmed by immunofluorescence confocal microscopy.

### Immunofluorescence confocal microscopy

**Figure 5:**
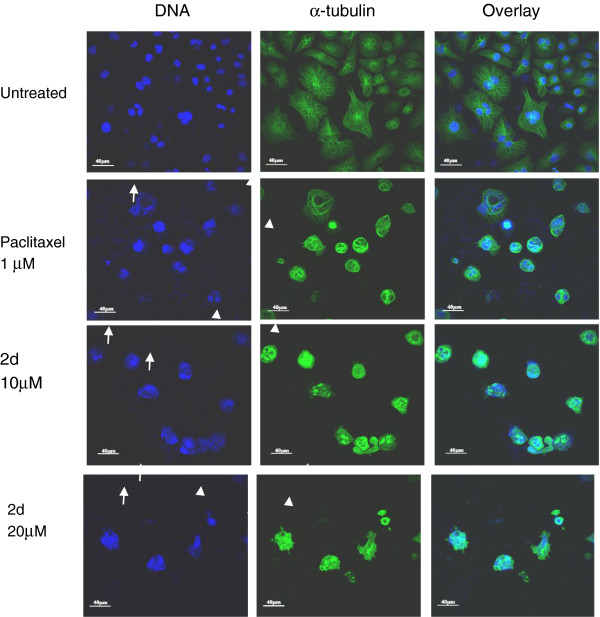
**Effects of paclitaxel and 2d on microtubules in THP-1 cells.** Cells were plated on coverslips and treated with 1 μM paclitaxel and 10 and 20 μM **2d** for 48 h. After treatment, cells were fixed and processed for immunofluorescence/confocal microscopy using monoclonal *α*-tubulin antibody and secondary antibody conjugated with Alexa-488 (Invitrogen, Carlsbad, USA). 4′,6-diamidino-2-phenylindole (DAPI) was used for nuclear staining. The untreated cells show normal nuclear morphology and microtubules. Paclitaxel treatment causes the condensation and fragmentation of nuclei due to apoptosis (arrow) and also causes loss of microtubules (arrowhead). **2d** treatment induces concentration-dependent increase in condensation and fragmentation of nuclei (arrow) due to apoptosis and also concentration-dependent loss of microtubules (arrowhead). Similar results were obtained in two separate experiments.

The immunofluorescence technique was used to evaluate the effect of **2d** on microtubules. The effect on microtubule structure was determined using confocal microscopy. Figure 5 shows representative images of microtubule structures and nuclei at 24 h of treatment. Minor changes in the microtubule structure were observed for 10 μM of **2d** at 24 h of treatment. At 30 μM, **2d** treatment at 24 h induced complete loss of microtubules. At the 48 h time point, cells treated with 10 and 20 μM **2d** showed a dose-dependent disruption and loss of microtubules compared to the control. Paclitaxel 1 μM was used as positive control. These results indicate that apoptosis induced by **2d** involves a severe loss of microtubule structure.

### Molecular docking of 2d

**Figure 6:**
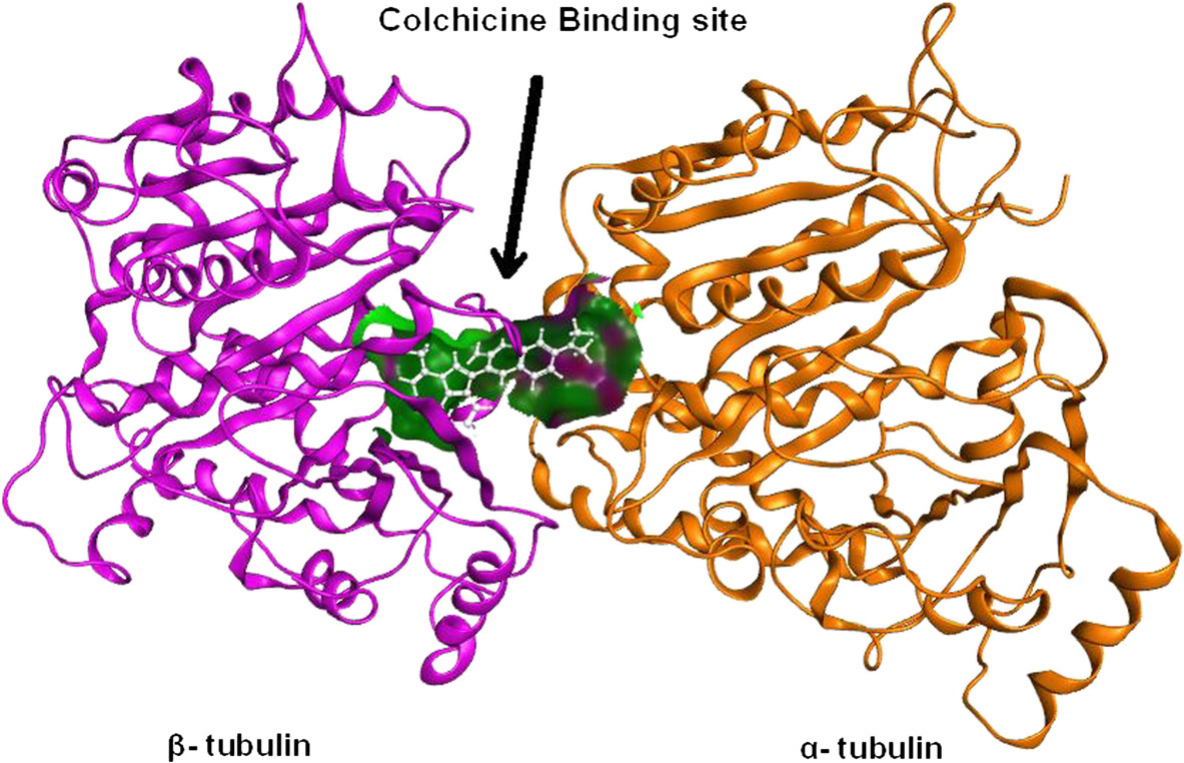
**Colchicine binding site at the interface between *α *and *β *subunits of tubulin.** The inhibitor **2d** is shown in silver color.

**Figure 7:**
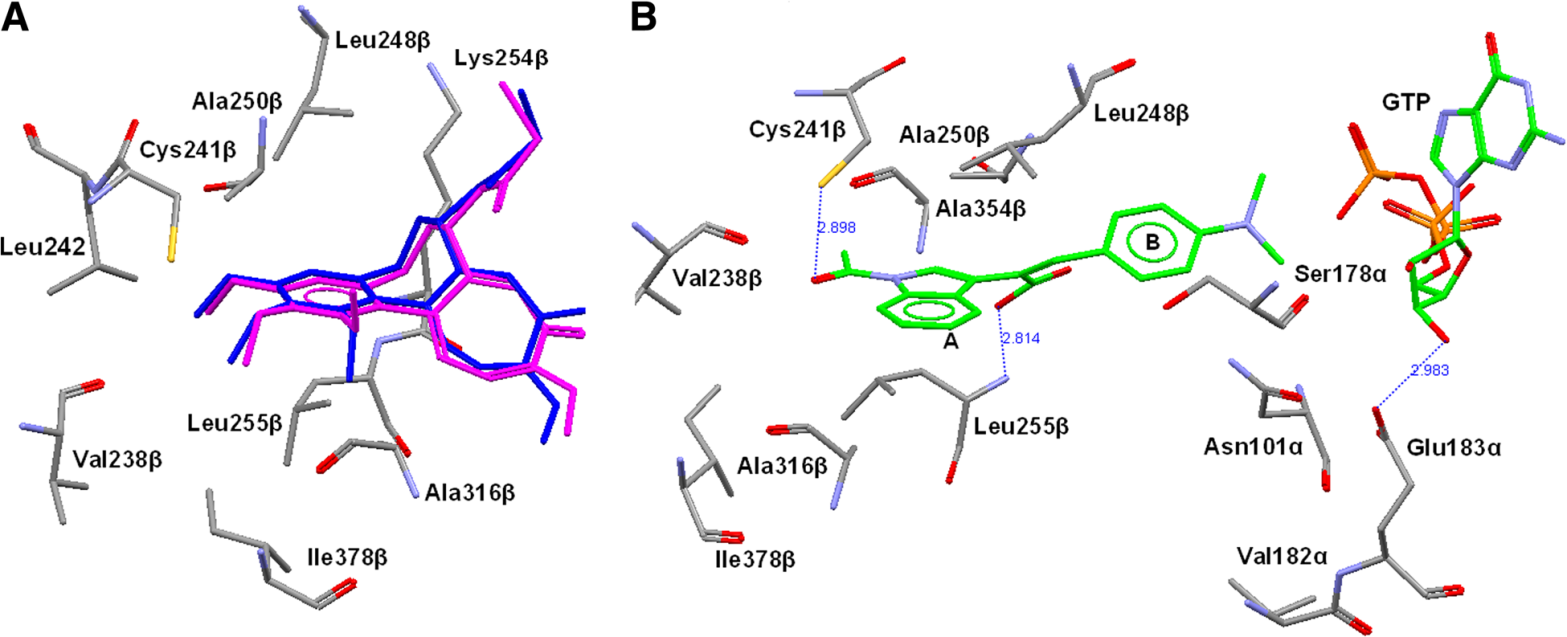
**Overlay of crystal structure and binding conformation. **(**A**) Overlay of crystal structure (blue) and docked (purple) conformation of colchicines, (**B**) binding conformation of **2d** (green) at the colchicine-binding site of tubulin.

In order to investigate the recognition process of these inhibitors at the colchicine-binding site of tubulin, a flexible docking study was performed on the most potent compound **2d** with the help of GOLD software [22]. The colchicine binding site encompasses the residues of both *α* and *β* subunits of tubulin (Figure 6). To validate the docking procedure for the prediction of the correct binding mode of inhibitor at the colchicine-binding site, the colchicine was extracted from the original X-ray structure [PDB:1SA0] [19,20] and re-docked using GOLD.21. The highest scoring conformation was selected and compared with X-ray structure conformation. The docked conformation of colchicine using GOLD was found to be similar with the original X-ray structure (Figure 7A). The root mean square deviation between the best scored conformers from docking and X-ray structure was found to be 0.72 Å. The compound **2d** was docked at the colchicine binding site of tubulin, and the best fit conformation was selected on the basis of Gold score and visual inspection. The Figure 7B shows the binding conformation of **2d** at the colchicine binding site. The compound **2d** gets stabilized at the colchicine-binding site of tubulin by hydrophobic and hydrogen bond interactions. The aromatic part of indole ring gets positioned in a hydrophobic cavity formed by Leu255*β*, Ala250*β*, Ala316*β*, Val318*β*, and Ala354*β*. The carbonyl oxygen (carbonyl group at indole) forms a hydrogen bond with sulfhydryl group of Cys241*β*. This is similar to the interaction of colchicines with the tubulin [25]. Another hydrogen bond was formed between the hydroxyl group of the carboxylic acid part of **2d** and nitrogen of Leu255*β*. These two hydrogen bonds play crucial role in stabilizing the conformation of **2d** at the colchicine-binding site. The unsaturated intermediate chain between rings A and B of **2d** is placed in close vicinity of the side-chain of Leu248*β* (Figure 7B). The ring B of **2d** is extended towards the *α*-chain of the tubulin. The docking study was helpful to find the binding conformation of **2d** at the colchicine binding site of tubulin.

### Experimental

The reagents were purchased from Sigma-Aldrich (MO, USA), LobaChemie (Mumbia, India), and Central Drug House (New Delhi, India) and used without further purification. All yields refer to isolated products after purification. Products were characterized by spectroscopic data (IR, [1] H NMR, and [13] C NMR spectra). The spectra were measured in DMSO-d_6_ relative to TMS (0.00 ppm). IR (KBr pallets) spectra were recorded on a Fourier transform infrared Thermo spectrophotometer (ThermoFisher Scientific Inc., Waltham, USA). Melting points were determined in open capillaries and were found to be uncorrected.

#### Typical experimental procedure for the synthesis combretastatin 2-(1-acetyl-1H-indole-3-yl)-3-(phenyl) propenoic analogues (2a to 2y)

A mixture of IAA (0.0057 mmol), substituted benzaldehyde (0.0057 mmol) and triethylamine (2 ml) in Ac_2_O (4 ml), was heated until the reaction was complete. After cooling, the reaction mixture was acidified with 35% aqueous HCl (6 ml) and kept at room temperature overnight when precipitates appeared as such or on dilution with water. The products were purified by column chromatography using silica gel of mesh size 60 to120.

### Characterization data

#### 2a. (Z)-2-(1-acetyl-1H-indol-3-yl)-3-(3,4,5-trimethoxyphenyl) propenoic acid

Mp: 177°C to 178°C; I.R. (KBr, cm^−1^): 1722 (C = O), 1681 (C = O, N-acetyl) 1636 (C = C), 1542 (C = C, phenyl ring), [1] H NMR (500 MHz, DMSO-d_6_, *δ*, TMS = 0, *J* = Hz): 8.44 (d, 1H, *J* = 8.25, H-7′), 8.03 (s, 1H, H-3), 7.90 (s, 1H, H-2′), 7.42 (1H, dd, *J* = 7.9 and 7.8, H-5′), 7.31 (dd, 1H, *J* = 7.7 and *J* = 7.8, H-6′), 7.25 (d, 1H, *J* = 8.0, H-4′), 6.64 (s, 1H, H-2″), 6.57 (s, 1H, H-6″), 3.63 (s, 3H, OMe) 3.34 (s, 6H, 2× OMe) 2.71 (s, 3H, N-acetyl); [13] C NMR (125 MHz, DMSO-d_6_, *δ*, TMS = 0, *J* = Hz): 169.40, 168.30, 152.20, 141.51, 138.38, 134.86, 129.35, 129.22, 125.95, 124.92, 123.46, 122.61, 119.84, 116.64, 115.97, 107.87, 59.96, 55.02, 23.70; MS: 418 (M + Na)^+^; Anal. calcd. For C_22_H_21_NO_6_: C, 66.83; H, 5.35; N, 3.54 found C: 66.73; H, 5.34; N, 3.34.

#### 2b. (Z)-2-(1-acetyl-1H-indol-3-yl)-3-(2,3,4-trimethoxyphenyl) propenoic acid

Mp: 210°C to 212°C; I.R. (KBr, cm^−1^): 1718 (C = O), 1668 (C = O, N-acetyl) 1637 (C = C), 1545 (C = C, phenyl ring); [1] H NMR (500 MHz, DMSO-d_6_, *δ*, TMS = 0, *J* = Hz): 8.36 (d, 1H, *J* = 8.3, H-7′), 8.16 (s, 1H, H-3) 7.84 (s, 1H, H-2′), 7.31 (dd, 1H, *J* = 7.3 and 8.0, H-6′), 7.14 (m, 2H, H-5′, 4′), 6.66 (d, 1H, *J* = 8.9, H-6″), 6.47 (d, 1H, *J* = 8.9, H-5″), 3.89 (s, 3H, OMe), 3.71 (s, 3H, OMe) 3.65 (s, 3H, OMe) 2.64 (s, 3H, N-acetyl); [13] C NMR (125 MHz, DMSO-d_6_, *δ*, TMS = 0, *J* = Hz): 169.40, 168.41, 154.55, 152.73, 141.32, 135.58, 134.94, 129.29, 126.02, 124.74, 123.99, 123.40, 122.00, 120.78, 119.52, 116.75, 116.03, 107.71, 61.52, 60.39, 55.74, 24.30; MS: 418 (M + Na)^+^; Anal. calcd. For C_22_H_21_NO_6_: C, 66.83; H, 5.35; N, 3.54 found C: 66.73; H, 5.34; N, 3.34.

#### 2c. (Z)-2-(1-acetyl-1H-indol-3-yl)-3-(3,4-dimethoxyphenyl) propenoic acid

Mp: 245°C to 247°C; I.R. (KBr, cm^−1^): 1712 (C = O), 1681 (C = O, N-acetyl) 1638 (C = C), 1554 (C = C, phenyl ring); [1] H NMR (500 MHz, DMSO-d_6_, *δ*, TMS = 0, *J* = Hz): 8.38 (d, 1H *J* = 8.3, H-7′), 7.96 (1H, s, H-3), 7.87 (1H, s, H-2′), 7.32 (dd, 1H, *J* = 7.3 and 7.2, H-6′), 7.16 (dd, 1H, *J* = 7.1, H-5) 7.12 (d, 1H, *J* = 7.6, H-4′) 6.97 (dd, 1H, *J* = 8.4 and 8.5, H-6″), 6.83 (d, 1H, *J* = 8.5, H-5″) 6.68 (d, 1H, *J* = 1.7, H-5″) 3.68 (s, 3H, OMe) 3.09 (s, 3H, OMe) 2.64 (s, 3H, N-acetyl); [13] C NMR (125 MHz, DMSO-d_6_, *δ*, TMS = 0, *J* = Hz): 168.36, 167.30, 148.80, 146.75, 140.50, 133.85, 128.20, 125.64, 124.80, 123.76, 123.63, 122.34, 119.73, 118.71, 115.79, 114.91, 111.40, 110.12, 54.27, 53.12, 22.70; MS: 388 (M + Na)^+^; Anal. calcd. For C_21_H_19_NO_5_: C, 69.03; H, 5.24; N, 3.83 found C: 69.0; H, 5.30; N, 3.74.

#### 2d. (Z)-2-(1-acetyl-1H-indol-3-yl)-3-(4-(dimethylamino) phenyl) propenoic acid

Mp: 246°C to 248°C; I.R. (KBr, cm^−1^), 3423 (NH), 1702 (C = O), 1682 (C = O, N-acetyl), 1659 (C = C), 1583 (C = C, phenyl ring); [1] H NMR (200 MHz, DMSO-d_6_, *δ*, TMS = 0, *J* = Hz): 8.43 (d, 1H *J* = 8.2, H-7′), 7.99 (s, 1H, H-3), 7.68 (s, 1H, H-2′), 7.14 to 7.30 (m, 5H, 5′, 6′, 4′, 2″, 6″), 6.20 (d, 2H, *J* = 8.9), 2.91 (s, 6H, 2× Me) 2.59 (s, 3H, N-acetyl); [13] C NMR (125 MHz, DMSO-d_6_, *δ*, TMS = 0, *J* = Hz); 169.62, 168.66, 150.83, 143.06, 135.55, 132.59, 132.11, 129.69, 124.88, 124.59, 123.43, 121.89, 120.30, 118.39, 117.08, 116.37, 111.24, 23.96; MS: 371 (M + Na)^+^; Anal. calcd. For C_21_H_20_N_2_O_3_: C, 72.4; H, 5.79; N, 8.04 found C: 72.1; H, 5.80; N, 8.14.

#### 2e. (Z)-2-(1-acetyl-1H-indol-3-yl)-3-(benzo[d][1,3]dixol-6-yl) propenoic acid

Mp: 243°C to 245°C; I.R. (KBr, cm^−1^): 1710 (C = O), 1683 (C = O, N-acetyl) 1637 (C = C), 1555 (C = C, phenyl ring); [1] H NMR (500 MHz, DMSO-d_6_, *δ*, TMS = 0, *J* = Hz): 8.38 (d, 1H *J* = 8.3, H-7′), 7.94 (s, 1H, H-3), 7.86 (s, 1H, H-2′), 7.33 (dd, 1H, *J* = 7.2 and 8.2, H-5′), 7.17 (dd, 1H, *J* = 7.7 and 7.3, H-6′) 7.11 (d, 1H, *J* = 7.8, H-4′) 6.93 (dd, 1H, *J* = 7.1 and 1.2, H-6″), 6.80 (d, 1H, *J* = 8.1, H-5″) 6.67 (s, 1H, H-2″) 5.90 (s, 2H, O-CH_2_-O) 2.65 (s, 3H, N-acetyl); [13] C NMR (125 MHz, DMSO-d_6_, *δ*, TMS = 0, *J* = Hz):169.50, 168.36, 148.26, 147.15, 141.30, 134.94, 129.12, 128.42, 126.17, 126.04, 124.85, 123.46, 121.42, 119.64, 116.51, 116.06, 108.76, 108.32, 101.35, 23.84; MS: 372 (M + Na)^+^; Anal. calcd. For C_20_H_15_NO_5_: C, 68.76; H, 4.33; N, 4.01 found C: 68.75; H, 4.41; N, 4.11. For further information, please refer to Additional file 1: Figures S1, Additional file 2: Figure S2, Additional file 3: Figure S3, Additional file 4: Figure S4, Additional file 5: Figure S5 and Additional file 6: Figure S6.

#### 2 f. (Z)-2-(1-acetyl-1H-indol-3-yl)-3-(2-nitrophenyl) propenoic acid

Mp: 209°C to 210°C; I.R. (KBr, cm^−1^): 1701 (C = O), 1683 (C = O, N-acetyl), 1655 (C = C), 1560 (C = C, phenyl ring); [1] H NMR (200 MHz, DMSO-d_6_, *δ*, TMS = 0, *J* = Hz): 8.39 (d, 1H *J* = 8.3, H-7′) 7.94 (s, 1H, H-3) 7.88 (s, 1H, H-2′) 7.6 to 7.70 (m, 4H, 3″, 4″, 5″, 6″) 7.39 (m, 3H, H-5′, 6′, 4′) 2.7 (s, 3H, N-acetyl); [13] C NMR (125 MHz, DMSO-d_6_, *δ*, TMS = 0, *J* = Hz): 168.58, 167.00 148.13, 138.99, 133.58, 133, 42, 130.76, 130.31, 129.04, 128.08, 125.31, 124.76, 124.57, 124.31, 123.36, 119.85, 119.57, 116.46, 23.90; MS: 373 (M + Na)^+^; Anal. calcd. For C_19_H_14_N_2_O_5_: C, 65.14; H, 4.03; N, 8.0 found C: 65.12; H, 4.03; N, 8.12.

#### 2 g. (Z)-2-(1-acetyl-1H-indol-3-yl)-3-(4-chlorophenyl) propenoic acid

Mp: 208°C to 209°C; I.R. (KBr, cm^−1^): 1713 (C = O), 1673 (C = O, N-acetyl), 1633 (C = C), 1580 (C = C, phenyl ring); [1] H NMR (200 MHz, DMSO-d_6_, *δ*, TMS = 0, *J* = Hz): 8.38 (d, 1H *J* = 8.9, H-7′), 7.99 (s, 1H, H-3) 7.58 to 7.61 (m, 4H, H-2′, 2″, 3″, 5″) 7.38 to 7.47 (m, 3H, H-5′, 6′, 6″), 7.26 (d, 1H, *J* = 7.9, 4′) 6.46 (s, 1H, NH, D_2_O exchangeable) 2.63 (s, 3H, N-acetyl); [13] C NMR (125 MHz, DMSO-d_6_, *δ*, TMS = 0, *J* = Hz): 168.67, 167.20, 142.78, 140.25, 135.51, 134.58, 133.49, 131.50, 129.53, 129.13, 128.55, 125.76, 125.15, 124.63, 123.59, 120.20, 119.98, 116.67, 23.71; MS: 362 (M + Na)^+^; Anal. calcd. For C_19_H_14_NO_3_Cl: C, 67.16; H, 4.15; N, 4.12 found C: 67.12; H, 4.23; N, 4.10.

#### 2 h. (Z)-2-(1-acetyl-1H-indol-3-yl)-3-(2,3-dichlorophenyl) propenoic acid

Mp: 225°C to 227°C; I.R. (KBr, cm^−1^): 1688 (C = O), 1624 (C = C), 1673 (C = O, N-acetyl), 1559 (C = C, phenyl ring); [1] H NMR (200 MHz, DMSO-d_6_, *δ*, TMS = 0, *J* = Hz): 8.43 (d, 1H *J* = 8.1, H-7′), 8.09 (s, 1H, H-3) 8.01 (s, 1H, H-2′), 7.51 to 7.6 (m, 4H, H-6′, 4″, 5″, 6″), 7.26 (d, 2H, *J* = 7.9, H-5′, 4′) 6.5 (s, 1H, NH, D_2_O exchangeable), 2.63 (s, 3H, N-acetyl); [13] C NMR (125 MHz, DMSO-d_6_, *δ*, TMS = 0 *J* = Hz): 167.67, 140.04, 135.17, 133.70, 132.63, 131.30, 127.49, 125.87, 122.99, 23.90; MS: 397 (M + Na)^+^; Anal. calcd. For C_19_H_13_NO_3_Cl_2_: C, 60.98; H, 3.50; N, 3.74 found C: 60.95; H, 3.50; N, 3.76.

#### 2i. (Z)-2-(1-acetyl-1H-indol-3-yl)-3-(2,4,5-trimethoxyphenyl) propenoic acid

Mp: 229°C to 230°C; I.R. (KBr, cm^−1^): 1701 (C = O), 1673 (C = O, N-acetyl), 1603 (C = C), 1514 (C = C, phenyl ring); [1] H NMR (200 MHz, DMSO-d_6_, *δ*, TMS = 0, *J* = Hz): 8.40 (d, 2H, *J* = 7.2, H-3, 7′), 7.96 (s, 1H, 2′), 7.31 to 7.43 (m, 3H, H-5′, 6′, 4′), 6.47 (s, 1H, D_2_O exchangeable), 6.40 (d, 2H, *J* = 7.6, H-5″, 6″), 3.89 (d, 6H, *J* = 11.3, 2× OMe), 2.9 (d, 3H, OMe) 2.73 (s, 3H, N-acetyl); [13] C NMR (100 MHz, DMSO-d_6_, *δ*, TMS = 0, *J* = Hz): 169.54, 168.59, 153.80, 151.37, 142.03, 136.41, 135.47, 129.52, 125.09, 124.92, 123.61, 120.56, 119.91, 118.16, 116.40, 114.80, 112.69, 96.29, 56.42, 55.82, 55.01, 23.94; MS: 418 (M + Na)^+^; Anal. calcd. For C_22_H_21_NO_6_: C, 66.83; H, 5.35; N, 3.54 found C: 66.85; H, 5.35; N, 3.30.

#### 2j. (Z)-2-(1-acetyl-1H-indol-3-yl)-3-(2-bromophenyl) propenoic acid

Mp: 199°C to 201°C; I.R. (KBr, cm^−1^): 1708 (C = O), 1666 (C = O, N-acetyl), 1622 (C = C), 1556 (C = C, phenyl ring); [1] H NMR (200 MHz, DMSO-d_6_, *δ*, TMS = 0,*J* = Hz): 8.43 (d, 1H, *J* = 8.7, H-7′), 7.98 (s, 1H, H-3), 7.48 (d, 1H, *J* = 9.0, H-2′), 7.32 to 7.20 (m, 3H, 6′, 3″, 6″), 7.01 (m, 4H, 5′, 4′, 4″, 6″), 2.64 (s, 3H, N-acetyl); [13] C NMR (125 MHz, DMSO-d_6_, *δ*, TMS = 0, *J* = Hz): 168.81, 168.68, 140.01, 136.90, 135.53, 133.01, 131.82, 129.75, 128.10, 125.32, 125.28, 125.20, 123.57, 122.11, 120.24, 116.65, 116.51, 24.00; MS: 407 (M + Na)^+^; Anal. calcd. For C_19_H_14_BrNO_3_: C, 59.39; H, 3.67; N, 3.65 found C, 59.24; H, 3.56; N, 3.45.

#### 2 k. (Z)-2-(1-acetyl-1H-indol-3-yl)-3-(naphthalene-2-yl) propenoic acid

Mp: 235°C to 237°C; I.R. (KBr, cm^−1^): 1708 (C = O), 1676 (C = O, N-acetyl), 1637 (C = C), 1560 (C = C, phenyl ring); [1] H NMR (200 MHz, DMSO-d_6_, *δ*, TMS = 0, *J* = Hz): 8.44 (d, 1H, *J* = 8.2, H-7′), 8.25 (s, 1H, H-3), 7.81 (s, 1H, H-2′), 7.68 (dd, 2H, *J* = 6.1 and 2.1, H-3″, 4″), 7.52 (1H, s, 1″) 7.44 to 7.30 (m, 4H, 8″, 7″, 6″, 4″), 7.11 (m, 3H, 5′, 6′, 4′) 2.6 (s, 3H, N-acetyl); [13] C NMR (125 MHz, DMSO-d_6_, *δ*, TMS = 0, *J* = Hz); 169.26, 168.73, 142.17, 135.56, 133.27, 132.87, 132.40, 131.33, 129.38, 128.37, 127.64, 127.48, 127.03, 126.32, 126.10, 125.18, 125.13, 123.68, 123.57, 120.38, 117.43, 116.49, 24; MS: 378 (M + Na)^+^; Anal. calcd. For C_23_H_17_NO_3_: C, 77.73; H, 4.82; N, 3.94 found C, 77.54; H, 4.64; N, 3.73.

#### 2 l. (Z)-2-(1-acetyl-1H-indol-3-yl)-3-(3-bromophenyl) propenoic acid

Mp: 226°C to 228°C; I.R. (KBr, cm^−1^): 1708 (C = O), 1676 (C = O, N-acetyl), 1622 (C = C), 1555 (C = C, phenyl ring); [1] H NMR (200 MHz, DMSO-d_6_, *δ*, TMS = 0, *J* = Hz): 8.41 (d, 1H, *J* = 7.6, H-7′), 7.95 (s, 1H, H-3) 7.75 (s, 1H, H-2′) 7.30 (dd, 2H, *J* = 7.1 and 3.3, H-6′), 7.21 to 7.00 (m, 4H, H-6′, 4″, 5″, 6″), 6.98 to 6.8 (m, 2H, H-5′, 4′), 2.65 (s, 3H, N-acetyl); [13] C NMR (100 MHz, DMSO-d_6_, *δ*, TMS = 0, *J* = Hz): 168.80, 168.63, 139.53, 137.02, 135.45, 132.82, 131.66, 129.76, 128.80, 128.09, 125.76, 125.41, 125.06, 123.47, 122.00, 120.19, 116.69, 116.40, 23.95; MS: 407 (M + Na)^+^; Anal. calcd. For C_19_H_14_BrNO_3_: C, 59.39; H, 3.67; Br, 20.80; N, 3.65 found C, 59.56; H, 3.54; N, 3.44.

#### 2 m. (Z)-2-(1-acetyl-1H-indol-3-yl)-3-(2-methoxyphenyl) propenoic acid

Mp: 229°C to 231°C; I.R. (KBr, cm^−1^): 1707 (C = O), ), 1677 (C = O, N-acetyl), 1603 (C = C), 1603 and 1450 (C = C, phenyl ring); [1] H NMR (200 MHz, DMSO-d_6_, *δ*, TMS = 0, *J* = Hz): 8.44 (d, 1H, *J* = 8.3, H-7′), 7.96 (s, 1H, H-3) 7.81 (s, 1H, H-2′), 7.27 (dd, 1H, *J* = 8.1 and 8.0, H-6′), 7.23 (d, 2H, *J* = 7.3, H-4″, 6″), 7.13 (dd, 2H, *J* = 7.6 and 2.9, 5′, 4′) 6.82 (dd, 2H, *J* = 7.4 and 7.6, H-3″, 5″) 2.64 (s, 3H, N-acetyl); [13] C NMR (125 MHz, DMSO-d_6_, *δ*, TMS = 0, *J* = Hz): 168.45, 160.00, 138.53, 136.19, 134.45, 130.87, 130.17, 129.53, 128.50, 127.89, 126.76, 125.51, 124.96, 123.45, 122.40, 121.09, 116. 23, 115.79, 24.10; MS: 378 (M + Na)^+^; Anal. calcd. For C_20_H_17_NO_4_: C, 71.63; H, 5.11; N, 4.18 found C, 71.63; H, 5.16; N, 4.26.

#### 2n. (Z)-2-(1-acetyl-1H-indol-3-yl)-3-(3-hydroxy-4-methoxyphenyl) propenoic acid

Mp: 235°C to 237°C; I.R. (KBr, cm^−1^): 2926 (O-H), 1702 (C = O), 1668 (C = O, N-acetyl), 1638 (C = C), 1504 (C = C, phenyl ring); [1] H NMR (200 MHz, DMSO-d_6_, *δ*, TMS = 0, *J* = Hz): 8.45 (d, 1H, *J* = 8.2, H-7′), 7.89 (s, 1H, H-3), 7.63 (s, 1H, H-2′), 7.29 (dd, 1H, *J* = 7.0 and 7.1, H-6′), 7.15 (dd, 2H, *J* = 7.2 and 7.2, H-5′, 4′), 6.79 to 7.0 (m, 3H, H-2″, 5″, 6″), 3.75 (s, 3H, CH_3_), 2.64 (s, 3H, N-acetyl ); [13] C NMR (100 MHz, DMSO-d_6_, *δ*, TMS = 0, *J* = Hz); 168.5, 168.46, 148.63, 145.65, 141.58, 135.03, 129.17, 127.01, 124.98, 124.31, 122.88, 122.82, 120.41, 119.64, 116.91, 116.15, 115.84, 110.86, 55.12, 23.70; MS: 374 (M + Na)^+^; Anal. calcd. For C_20_H_17_NO_5_: C, 68.37; H, 4.88; N, 3.99 found C, 68.21; H, 4.62; N, 4.13.

#### 2o. (Z)-2-(1-acetyl-1H-indol-3-yl)-3-(4-hydroxy-3-methoxyphenyl) propenoic acid

Mp: 212°C to 213°C; I.R. (KBr, cm^−1^): 2930 (OH), 1701 (C = O), 1681 (C = O, N-acetyl), 1599 (C = C), 1451 (C = C, phenyl ring); [1] H NMR (200 MHz, DMSO-d_6_, *δ*, TMS = 0, *J* = Hz): 9.22 (s, 1H, OH, D_2_O exchangeable), 8.43 (d, 1H, *J* = 8.2, H-7′), 8.03 (s, 1H, H-3), 7.83 (s, 1H, H-2′) 7.30 (dd, 2H, *J* = 7.2 and 7.3, H-5′, 6′) 6.90 (s, 1H, H-6″), 6.82 to 6.70 (m, 3H, 4′, 2″, 3″), 3.15 (s, 3H, OMe) 2.59 (s, 3H, N-acetyl ); [13] C NMR (125 MHz, DMSO-d_6_, *δ*, TMS = 0, *J* = Hz): 167.88, 167.50, 147.05, 145.72, 141.15, 134.05, 128.11, 124.56, 124.25, 123.93, 123.70, 122.20, 119.07, 118.36, 116.37, 113.91, 111.62, 53.37, 22.70; MS: 374 (M + Na)^+^; Anal. calcd. For C_20_H_17_NO_5_: C, 68.37; H, 4.88; N, 3.99 found C, 68.22; H, 4.71; N, 4.03.

#### 2p. (Z)-2-(1-acetyl-1H-indol-3-yl)-3-(2,5-dimethoxyphenyl) propenoic acid

Mp: 246°C to 248°C; I.R. (KBr, cm^−1^) 1710 (C = O), 1671 (C = O, N-acetyl), 1611 (C = C), 1452 (C = C, phenyl ring); [1] H NMR(200 MHz, DMSO-d_6_, *δ*, TMS = 0, *J* = Hz): 8.79 (d, 1H, *J* = 7.2, H-7′), 8.37 (s, 1H, H-3), 8.02 (s, 1H, H-2′), 7.44 (dd, 1H, *J* = 8.1 and 8.0, H-6′), 7.17 to 7.20 (m, 2H, H-5′, 4′), 6.90 (d, 1H, *J* = 2.5, H-2″), 6.88 (d, 1H, *J* = 7.4, H-5″), 6.77 (d, 1H, *J* = 7.4, H-6″) 3.50 (s, 3H, OMe), 3.11 (s, 3H, OMe) 2.71 (s, 3H, N-acetyl ); [13] C NMR (125 MHz, DMSO-d_6_, *δ*, TMS = 0, *J* = Hz): 167.19, 167.00, 155.04, 150.65, 150.61, 134.72, 133.53, 128.73, 127.69, 124.00, 123.18, 122.07, 121.75, 118.45, 115.47, 115.31, 114.53, 112.28, 112.11, 110.27, 22.20; MS: 388 (M + Na)^+^; Anal. calcd. For C_21_H_19_NO_5_: C, 69.03; H, 5.24; N, 3.83 found C: 69.03; H, 5.37; N, 3.64.

#### 2q. (Z)-2-(1-acetyl-1H-indol-3-yl)-3-(furan-2-yl) propenoic acid

Mp: 231°C to 232°C; I.R. (KBr, cm^−1^): 1711 (C = O), 1671 (C = O, N-acetyl), 1640 (C = C), 1564 (C = C, phenyl ring); [1] H NMR (200 MHz, DMSO-d_6_, *δ*, TMS = 0, *J* = Hz): 8.49 (d, 1H, *J* = 8.8, H-7′), 8.00 (s, 1H, H-3) 7.49 (s, 1H, H-2′) 7.35 (d, 1H, *J* = 5.3, H-2″), 7.27 to 7.10 (m, 3H, H-5′, 6′, 4′), 6.20 (dd, 2H, *J* = 3.4 and 3.5, H-3″, 4”) [13] C NMR (100 MHz, DMSO-d_6_, *δ*, TMS = 0, *J* = Hz): 168.50, 167.42, 149.78, 142.94, 141.40, 136.61, 134.49, 130.82, 126.00, 125.20, 124.86, 123.28, 122.77, 121.00, 120.42, 111.60, 24.30; MS: 318 (M + Na)^+^; Anal. calcd. For C_17_H_13_NO_4_: C: 69.15; H, 4.44; N, 4.74 found C: 69.11; H, 4.53; N, 4.54.

#### 2r. (2Z,4E)-2-(1-acetyl-1H-indol-3-yl)-5-phenylpenta-2, 4-dienoic acid

Mp: 232°C to 234°C; I.R. (KBr, cm^−1^): 1725 (C = O), 1671 (C = O, N-acetyl), 1610 (C = C), 1449 (C = C, phenyl ring); [1] H NMR (200 MHz, DMSO-d_6_, *δ*, TMS = 0, *J* = Hz): 8.52 (d, 1H, *J* = 8.4, H-7′), 7.91 (s, 1H, H-3), 7.52 (s, 1H, H-2′), 7.41 to 7.37 (m, 3H, 5′, 6′, 4′), 7.29 to 7.0 (m, 5H, 2″, 3″, 4″, 5″, 6″), 7.03 (dd, 1H, *J* = 15.5 and 15, H-4), 6.82 (d, 1H, *J* = 14, H-5), 2.68 (s, 3H, N-acetyl); [13] C NMR (100 MHz, DMSO-d_6_, *δ*, TMS = 0, *J* = Hz): 170.89, 168.63, 144.60, 142.27, 135.93, 135.51, 130.14, 129.42, 128.82, 127.46, 125.67, 125.52, 124.86, 123.94, 122.09, 120.23, 116.69, 116.08, 24.00; MS: 318 (M + Na)^+^; Anal. calcd. For C_17_H_13_NO_4_: C: 76.12; H, 5.17; N, 4.23 found C: 76.23; H, 5.06; N, 4.33.

#### 2 s. (Z)-2-(1-acetyl-1H-indol-3-yl)-3-(4-nitrophenyl) propenoic acid

Mp: 222°C to 223°C; I.R. (KBr, cm^−1^): 1684 (C = O), 1671 (C = O, N-acetyl), 1629 (C = C), 1601 and 1450 (C = C, phenyl ring); [1] H NMR (200 MHz, DMSO-d_6_, *δ*, TMS = 0, *J* = Hz): 8.41 (d, 1H, *J* = 8.2, H-7′), 8.32 (s, 1H, H-3), 8.26 (d, 1H, *J* = 4.7, H-2″), 7.58 to 7.63 (m, 2H, H-2′, 4″), 7.39 (d, 2H, H-4″, 5″), 7.31 to 7.11 (m, 3H, H-5′, 6′, 4′), 2.48 (s, 3H, N-acetyl); [13] C NMR (100 MHz, DMSO-d_6_, *δ*, TMS = 0, *J* = Hz): 168.00, 167.62, 148.78, 141.94, 136.61, 134.49, 130.82, 124.86, 123.28, 122.77, 24.00; MS: 373 (M + Na)^+^; Anal. calcd. For C_19_H_14_N_2_O_5_: C, 65.14; H, 4.03; N, 8.0 found C: 65.17; H, 4.12; N, 8.05.

#### 2 t. (Z)-2-(1-acetyl-1H-indol-3-yl)-3-(4-fluorophenyl) propenoic acid

Mp: 228°C to 229°C; I.R. (KBr, cm^−1^): 1711 (C = O), 1679 (C = O, N-acetyl), 1629 (C = C), 1599 and 1450 (C = C, phenyl ring); [1] H NMR (200 MHz, DMSO-d_6_, *δ*, TMS = 0, *J* = Hz): 8.37 (d, 1H, *J* = 8.2, H-7′), 8.00 (s, 1H, H-3), 7.87 (s, 1H, H-2′) 7.33 (m, 3H, 5′, 6′, 4′), 7.07 to 6.9 (m, 4H, 2″, 3″, 5″, 6″), 2.48 (s, 3H, N-acetyl); [13] C NMR (100 MHz, DMSO-d_6_, *δ*, TMS = 0, *J* = Hz): 169.90, 168.63, 151.00, 140.53, 137.02, 135.45, 132.62, 132.66, 129.36, 126.60, 125.76, 123.47, 122.00, 120.19, 116.69, 116.40, 119.90, 115.70, 24.20: 346 (M + Na)^+^; Anal. calcd. For C_19_H_14_FNO_3_: C, 70.58; H, 4.36; N, 4.33 found C, 70.52; H, 4.31; N, 4.23.

#### 2u. (Z)-2-(1-acetyl-1H-indol-3-yl)-3-(3-chlorophenyl) propenoic acid

Mp: 228°C to 229°C; I.R. (KBr, cm^−1^): 1708 (C = O), 1665 (C = O, N-acetyl), 1623 (C = C), 1449 (C = C, phenyl ring); [1] H NMR (200 MHz, DMSO-d_6_, *δ*, TMS = 0, *J* = Hz): 8.38 (d, 1H, *J* = 8.2, H-7′), 7.99 (s, 1H, H-3), 7.89 (s, 1H, H-2′), 7.31 to 7.50 (m, 6H, 5′, 6′, 4′, 3″, 4″, 5″), 2.48 (s, 3H, N-acetyl); [13] C NMR (125 MHz, DMSO-d_6_, *δ*, TMS = 0, *J* = Hz): 169.90, 168.63, 151.00, 140.53, 137.02, 135.45, 132.62, 132.66, 129.36, 126.60, 125.76, 123.47, 122.00, 120.19, 116.69, 116.40, 119.90, 115.70, 19.30; MS: 362 (M + Na)^+^; Anal. calcd. For C_19_H_14_NO_3_Cl: C, 67.16; H, 4.15; N, 4.12 found C: 67.23; H, 4.11; N, 4.02.

#### 2v. (Z)-2-(1-acetyl-1H-indol-3-yl)-3-(2-chlorophenyl) propenoic acid

Mp: 189°C to 190°C; I.R. (KBr, cm^−1^): 1720 (C = O), 1685 (C = O, N-acetyl), 1621 (C = C), 1450 (C = C, phenyl ring); [1] H NMR (200 MHz, DMSO-d_6_, *δ*, TMS = 0, *J* = Hz): 8.38 (d, 1H, *J* = 7.32, H-7′), 8.10 (s, 1H, H-3), 7.91 (d, 1H, *J* = 6.8, H-2′), 7.71 (d, 1H, H-3″), 7.43 to 7.30 (m, 3H, 5′, 6′, 4′), 6.80 to 6.9 (m, 3H, 4″, 5″, 6″), 2.62 (s, 3H, N-acetyl ); [13] C NMR (100 MHz, DMSO-d_6_, *δ*, TMS = 0, *J* = Hz): 169.80, 168.20, 139.53, 137.02, 135.45, 132.62, 132.66, 129.80, 126.60, 125.76, 123.47, 122.00, 119.00, 116.69, 116.40, 115.7, 24.20; MS: 362 (M + Na)^+^; Anal. calcd. For C_19_H_14_NO_3_Cl: C, 67.16; H, 4.15; N, 4.12 found C: 66.27; H, 4.11; N, 4.32.

#### 2w. (Z)-2-(1-acetyl-1H-indol-3-yl)-3-(naphthalene-1-yl) propenoic acid

Mp: 190°C to 192°C; I.R. (KBr, cm^−^1): 1703 (C = O), 1680 (C = O, N-acetyl), 1615 (C = C), 1449 (C = C, phenyl ring); [1] H NMR (200 MHz, DMSO-d_6_, *δ*, TMS = 0, *J* = Hz): 8.23 (d, 1H, *J* = 7.9, H-7′), 8.16 (s, 1H, H-3), 8.00 (d, 1H, *J* = 6.2, H-2′), 7.64 to 7.3 (m, 7H, 2″, 3″, 4″, 5″, 6″, 7″, 8″) 6.93 to 6.80 (m, 3H, 5′, 6′, 4′), 2.60 (s, 3H, N-acetyl); [13] C NMR (100 MHz, DMSO-d_6_, *δ*, TMS = 0, *J* = Hz); 168.50, 167.88, 140.66, 139.18, 135.56, 133.27, 132.87, 132.40, 131.20, 129.18, 127.64, 126.32, 126.10, 125.18, 123.68, 123.57, 120.38, 117.43, 116.49, 24.20; 378 (M + Na)^+^; Anal. calcd. For C_23_H_17_NO_3_: C, 77.73; H, 4.22; N, 3.94 found C, 77.45; H, 4.32; N, 3.83.

#### 2x. (Z)-2-(1-acetyl-1H-indol-3-yl)-3-(4-bromophenyl) propenoic acid

Mp: 220°C to 222°C; I.R. (KBr, cm^−1^): 1711 (C = O), 1676 (C = O, N-acetyl), 1632 (C = C), 1565 (C = C, phenyl ring); [1] H NMR (200 MHz, DMSO-d_6_, *δ*, TMS = 0, *J* = Hz): 8.41 (d, 1H, *J* = 7.6, H-7′), 7.95 (s, 1H, H-3) 7.75 (s, 1H, H-2′) 7.30 (dd, 2H, *J* = 7.1 and 3.3, H-6′), 7.21 to 7.10 (m, 4H, H-6′, 3″, 5″, 6″), 6.98 to 6.80 (m, 2H, H-5′, 4′), 2.68 (s, 3H, N-acetyl); [13] C NMR (125 MHz, DMSO-d_6_, *δ*, TMS = 0, *J* = Hz): 168.00, 168.53, 139.53, 137.02, 135.45, 132.82, 131.66, 129.76, 128.80, 128.09, 125.76, 125.41, 125.06, 123.47, 122.00, 120.19, 116.69, 116.40, 23.00: 407 (M + Na)^+^;Anal. calcd. For C_19_H_14_BrNO_3_: C, 59.39; H, 3.67; N, 3.65; found C, 59.22; H, 3.4; N, 3.45.

#### 2y. 3-(N-acetyl-1H-indol-3-yl)-8-acetoxy-2H-chromen-2-one

Mp: 110°C to 112°C; I.R. (KBr, cm^−1^): 1763 (C = O, lactone) 1710 (C = O, O-acetyl), 1703 (C = O, N-acetyl), 1684 (C = O), 1543 and 1453 (C = C, phenyl ring); [1] H NMR (200 MHz, DMSO-d_6_, *δ*, TMS = 0, *J* = Hz): 8.51 (s, 1H, H-4), 8.45 (d, 1H, *J* = 7.0, H-7′), 8.35 (s, 1H, H-2′), 7.95 (dd, 1H, *J* = 5.2 and 7.2, H-6′) 7.95 (dd, 1H, *J* = 5.2 and 7.2, H-5′), 7.48 to 7.38 (m, 4H, H-4′, 5′, 6′, 7′), 2.67 (s, 3H, N-acetyl ), 2.67 (s, 3H, O-acetyl ); [13] C NMR (125 MHz, DMSO-d_6_, *δ*, TMS = 0, *J* = Hz): 170.00, 168.93, 158.82, 148.78, 144.48, 139.62, 137.20, 135.50, 128.3, 128.27, 126.55, 125.70, 125.40, 125.00, 124.29, 12.05, 121.00, 120.90, 116.49, 114.98, 24.30, 20.81; MS: 386 (M + Na)^+^; Anal. calcd. For C_20_H_13_NO_6_: C, 66.12; H, 3.61; N, 3.86; found C, 66.24; H, 3.42; N, 3.88

### Biological evaluation

*In vitro* cytotoxicity against five human cancer cell lines was determined using 96-well tissue culture plate [26]. The cells were allowed to grow in carbon dioxide incubator (37°C) for 24 h. Test materials in complete growth medium (100 μl) were added after 24 h of incubation to the wells containing cell suspension. The plates were further incubated for 48 h in a carbon dioxide incubator. The cell growth was stopped by gentle layering trichloroacetic acid (50%, 50 μl) on top of the medium in all the wells. The plates were incubated at 4°C for 1 h to fix the cells attached to the bottom of the wells. The liquid of all the wells was gently pipetted out and discarded. The plates were washed five times with distilled water to remove trichloroacetic acid, growth medium low-molecular weight metabolites, serum proteins, etc. and air-dried. The plates were stained with sulforhodamine B dye (0.4% in 1% acetic acid, 100 μl) for 30 min. The plates were washed five times with 1% acetic acid and then air-dried [27]. The adsorbed dye was dissolved in Tris–HCl buffer (100 μL, 0.01 M, pH 10.4), and the plates were gently stirred for 10 min on a mechanical stirrer. The optical density (OD) was recorded on ELISA reader at 540 nm. The cell growth was determined by subtracting the mean OD value of respective blank from the mean OD value of the experimental set. Percent growth in presence of test material was calculated considering the growth in the absence of any test material as 100%, and in turn, percent growth inhibition in presence of test material was calculated.

## Conclusions

A series of novel combretastatin analogues have been synthesized by condensation of indole-3-acetic acid with different substituted aldehydes, and structures were established by various spectral techniques like IR, MS, and NMR ([1] H, [13] C, HMBC, and HSQC). Configuration was established by lactone formation when hydroxyl group was present on second position of aldehyde. Further, it was confirmed by nOe (H-4′ intensity increased on double irradiation of H-3). Compounds were evaluated for anticancer activity. The effect on microtubule structure was determined using confocal microscopy dose-dependent disruption, and loss of microtubules indicates apoptosis by **2d**. Binding of compound at colchicine binding site was established by molecular docking study. It was helpful to rationalize the inhibitory activity of these inhibitors. The carbonyl oxygen (carbonyl group at indole) forms a hydrogen bond with sulfhydryl group of Cys241*β*. This is similar to the interaction of colchicines with the tubulin. Another hydrogen bond was formed between the hydroxyl group of the carboxylic acid part of **2d** and nitrogen of Leu255*β*. These two hydrogen bonds play a crucial role in stabilizing the conformation of **2d** at the colchicine binding site. The activity of the synthesized compounds indicates that these can be promising anticancer drugs. To confirm their potency, further *in vivo* experiment will be required to address the anticancer property in cancer chemotherapy.

## Competing interests

The authors declare that they have no competing interests.

## Supplementary Material

Additional file 1 **Compound 2e. **C13 spectrum. Click here for file

Additional file 2 **Compound 2e.** DEPT spectrum. Click here for file

Additional file 3 **Compound 2e. **H1 NMR spectrum. Click here for file

Additional file 4 **Compound 2e.** HMBC spectrum. Click here for file

Additional file 5 **Compound 2e. **Expanded HMBC spectrum. Click here for file

Additional file 6 **Compound 2e.** Expanded HSQC spectrum. Click here for file

## References

[B1] Jordan A Hadfield JA Lawrence NJ McGown AT Tubulin as a target for anticancer drugs: agent which interact with mitotic spindle Inc Med Res Rev 1997 18 259 296 10.1002/(sici)1098-1128(199807)18:4<259::aid-med3>3.0.co;2-u 9664292

[B2] Hadfield JA Ducki S Hirst N McGown AT Tubulin and microtubules as target for anticancer drugs Progress in Cell Cycle Res 2003 5 309 325 14593726

[B3] Meng F Cai X Duan J Matteuccki MG Hart CP A novel class of tubulin inhibitors that exhibit potent antiproliferation and in vitro vessel-disrupting activity Canc Chemoth Pharmacol 2008 61 953 963 10.1007/s00280-007-0549-x 17639393

[B4] Toso RJ Jordan MA Farrel KW Matsumoto B Wilson L Kinetic stabilization of microtubule dynamic instability in vitro by vinblastine Biochem 1993 32 1285 1293 10.1021/bi00056a013 8448138

[B5] Wu R Ding W Liu T Zhu H Hu Y Yang B He Q XNO_5_, a novel synthesized microtubule inhibitor, exhibits potent activity against human carcinoma cells in vitro Canc Lett 2009 285 13 22 10.1016/j.canlet.2009.04.042 19647933

[B6] Mitchison T Kirschner M Dynamic instability of microtubule growth Nature 1984 312 237 242 10.1038/312237a0 6504138

[B7] Downing KH Nogales E New insights into microtubule structure and function from the atomic model of tubulin Eur Biophys J 1998 27 431 436 10.1007/s002490050153 9760724

[B8] Ouyang X Piatnitski EL Pattaropong V Chen X He HY Kiselyov AS Valankar A Kwakami J Labelle M Smith L Lohman J Lee SP Malikzay A Fleming J Gerlak J Wang Y Rosler RL Zhou K Mitelman S Camara M Surguladze D Boody JF Tuma MC Oxadiazole derivatives as novel class of antimitotic agents: synthesis, inhibition of tubulin polymerization and activity in tumor cell lines Bioorg Med Chem Lett 2006 16 1191 1196 10.1016/j.bmcl.2005.11.094 16377187

[B9] Tron GC Pirali T Sorba G Pagliai F Busacca S Genazzani AA Medicinal chemistry of combretastatin A4: present and future directions J Med Chem 2006 49 3033 3044 10.1021/jm0512903 16722619

[B10] Woods JA Hadfield JA Pettit GR Fox BW McGown AT The interaction with tubulin of a series of stilbenes based on combretastatin A-4 Br J Canc 1995 71 705 711 10.1038/bjc.1995.138 PMC2033763 7710932

[B11] McGown AT Fox BW Structural and biochemical comparison of the antimitotic agents colchicine, combretastatin A4 and amphethinile Anticancer Drug Des 1989 3 249 254 2930627

[B12] Petit GR Rhodes MR Herald DL Hamel E Schmidt JM Petit RK Antineoplastic agents, 445, synthesis and evaluation of structural modifications of (Z), and (E)-combretastatin A-4 J Med Chem 2005 48 4087 4099 10.1021/jm0205797 15943482

[B13] Kaffy J Pontikis R Carrz D Croisy A Monneret C Florent JC Isoxazole-type derivatives related to combretastatin A-4, synthesis and biological evaluation Bioorg Med Chem 2006 14 4067 4077 10.1016/j.bmc.2006.02.001 16510288

[B14] Pettit RK Pettit GR Hamel E Hogan F Moser BR Wolf S Pon S Chapuis JC Schmidt JM E-combretastatin and E-resveratrol structural modifications: antimicrobial and cancer cell growth inhibitory β-E-nitrostyrenes Bioorg Med Chem 2009 17 6606 6612 10.1016/j.bmc.2009.07.076 19709889

[B15] Gupta S Bhattacharyya B Antimicrotubular drugs binding to vinca domain of tubulin Mol Cell Biochem 2003 253 41 47 10.1023/A:1026045100219 14619954

[B16] Cragg GM Newman DJ Ethnopharmacology 2006 Oxford: EOLSS

[B17] Cragg GM Newman DJ Anticancer agents from natural products CRCnetBASE 2011 2 699 728

[B18] Pinard PV Wang F Burd B Angeletti RH Horwitz SB Orr GA Direct analysis of tubulin expression in cancer cell lines by electrospray ionization mass spectrometry Biochem 2003 42 12019 12027 10.1021/bi0350147 14556633

[B19] Enroth C Eger BT Okamoto K Nishino T Nishino T Pai EF Crystal structure of bovine milk xanthene dehydrogenase: structure based mechanism of conversion Proc Natl Acad Sci USA 2000 97 10723 10.1073/pnas.97.20.10723 11005854 PMC27090

[B20] Ravelli RB Gigant B Curmi PA Jourdain I Lachkar S Sobel A Knossow M Insight into tubulin regulation from a complex with colchicine and a stathmin-like domain Nature 2004 428 198 202 10.1038/nature02393 15014504

[B21] PerkinElmer, Inc ChemDraw Ultra 6.0 and Chem3D Ultra 2000 Cambridge, USA: Cambridge Soft Corporation

[B22] CCDC GOLD 5.0.1 2011 Cambridge, UK: Cambridge Crystallographic Data Centre

[B23] Cushman MS Layfayette W Hamel E Stilbene derivatives as anticancer agents 1995 US Patent 5,430,062, Bethesda, 4 July 1995

[B24] Leake PH Synthesis of phenanthrene Chem Rev 1956 56 27 10.1021/cr50007a002

[B25] Sylvie D Grant M Ben G Simon A Jérémie FDC Elizabeth B Jim N James P Nicholas JL Combretastatin-like chalcones as inhibitors of microtubule polymerisation. Part 2: Structure-based discovery of alpha-aryl chalcones Bioor Med Chem 2009 17 7711 7722 10.1016/j.bmc.2009.09.044 19837594

[B26] Monks A Scudiero D Skehan P Shoemaker R Paull K Vistica D Hose C Langley J Cronise P Vaigro-Wolff A Feasibility of a high-flux anticancer drug screen using a diverse panel of cultured human tumor cell lines J Natl Canc Inst 1991 83 757 766 10.1093/jnci/83.11.757 2041050

[B27] Skehan P Storeng R Scudiero D Monks A McMahon J Vistica D Warren JT Bokesch H Kenney S Boyd MR New colorometric cytotoxicity assay for anticancer-drug screening J Natl Canc Inst 1990 82 1107 1112 10.1093/jnci/82.13.1107 2359136

