# Supplementary figures and images for: Novel indole-bearing combretastatin analogues as tubulin polymerization inhibitors
Source: Org Med Chem Lett. 2013 Mar 3;3:3. doi: 10.1186/2191-2858-3-3 (PMC3599526; doi:10.1186/2191-2858-3-3)

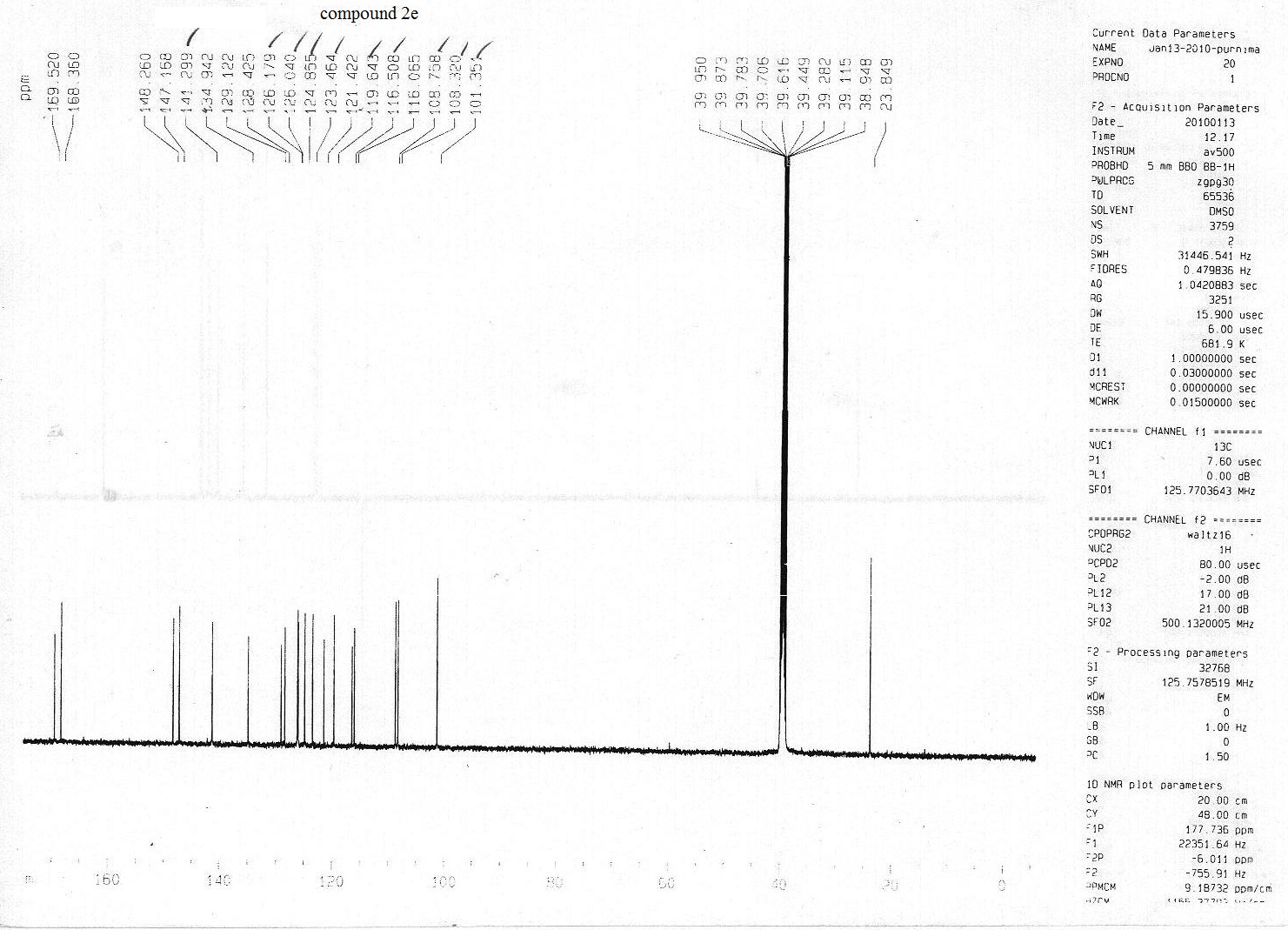

Supplement: Additional file 1 — Compound 2e. C13 spectrum. [file 2191-2858-3-3-S1.jpeg]

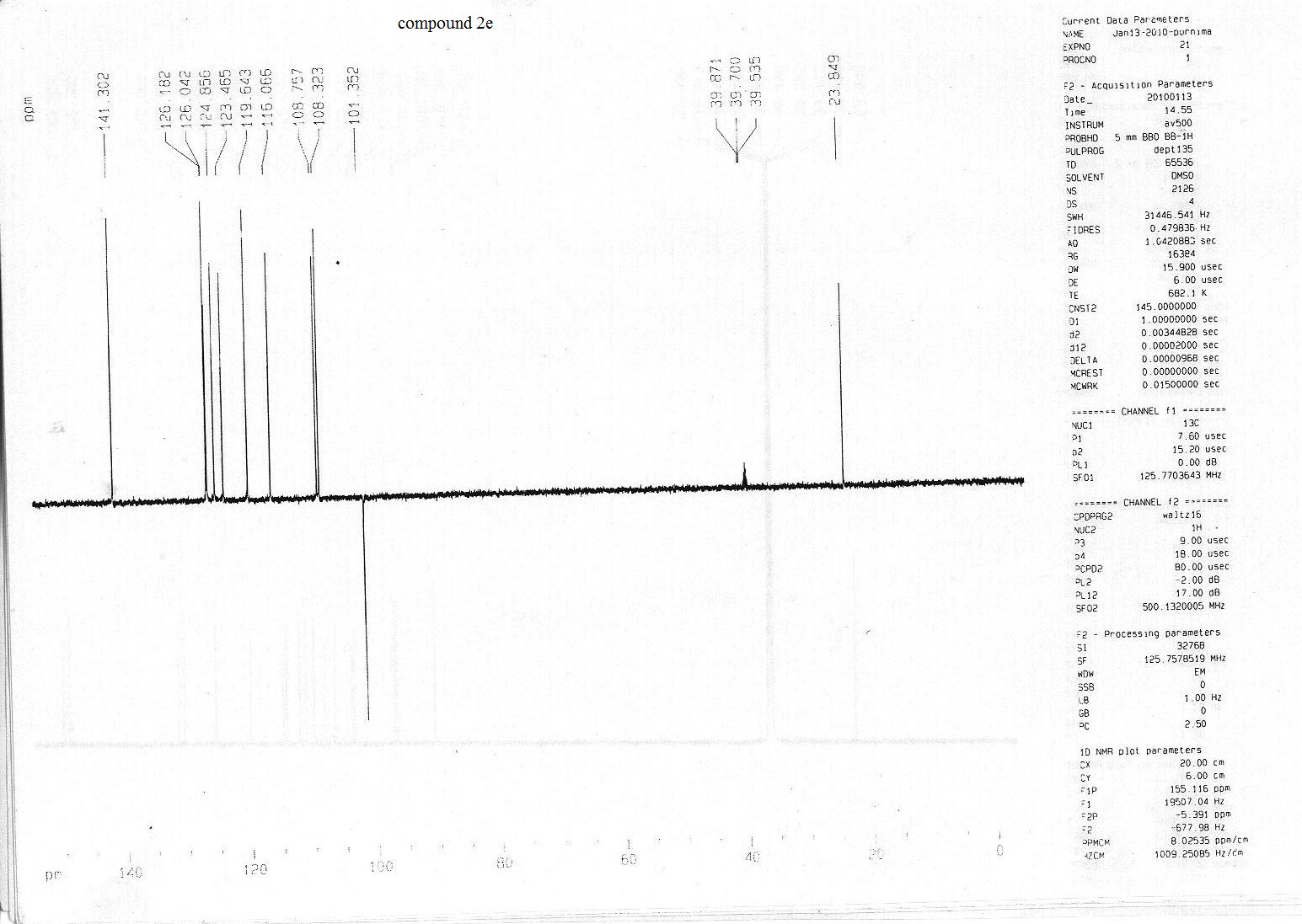

Supplement: Additional file 2 — Compound 2e. DEPT spectrum. [file 2191-2858-3-3-S2.jpeg]

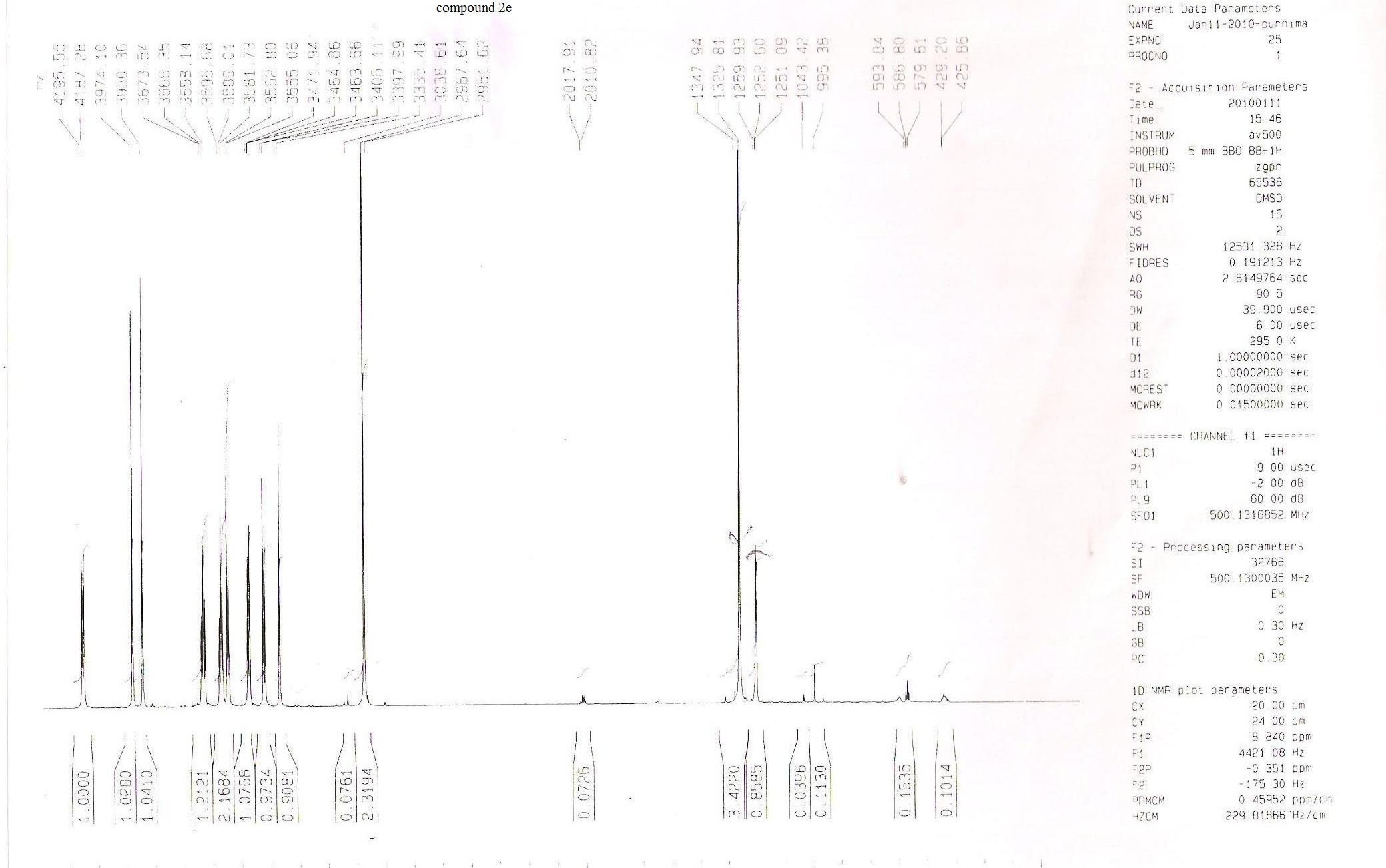

Supplement: Additional file 3 — Compound 2e. H1 NMR spectrum. [file 2191-2858-3-3-S3.jpeg]

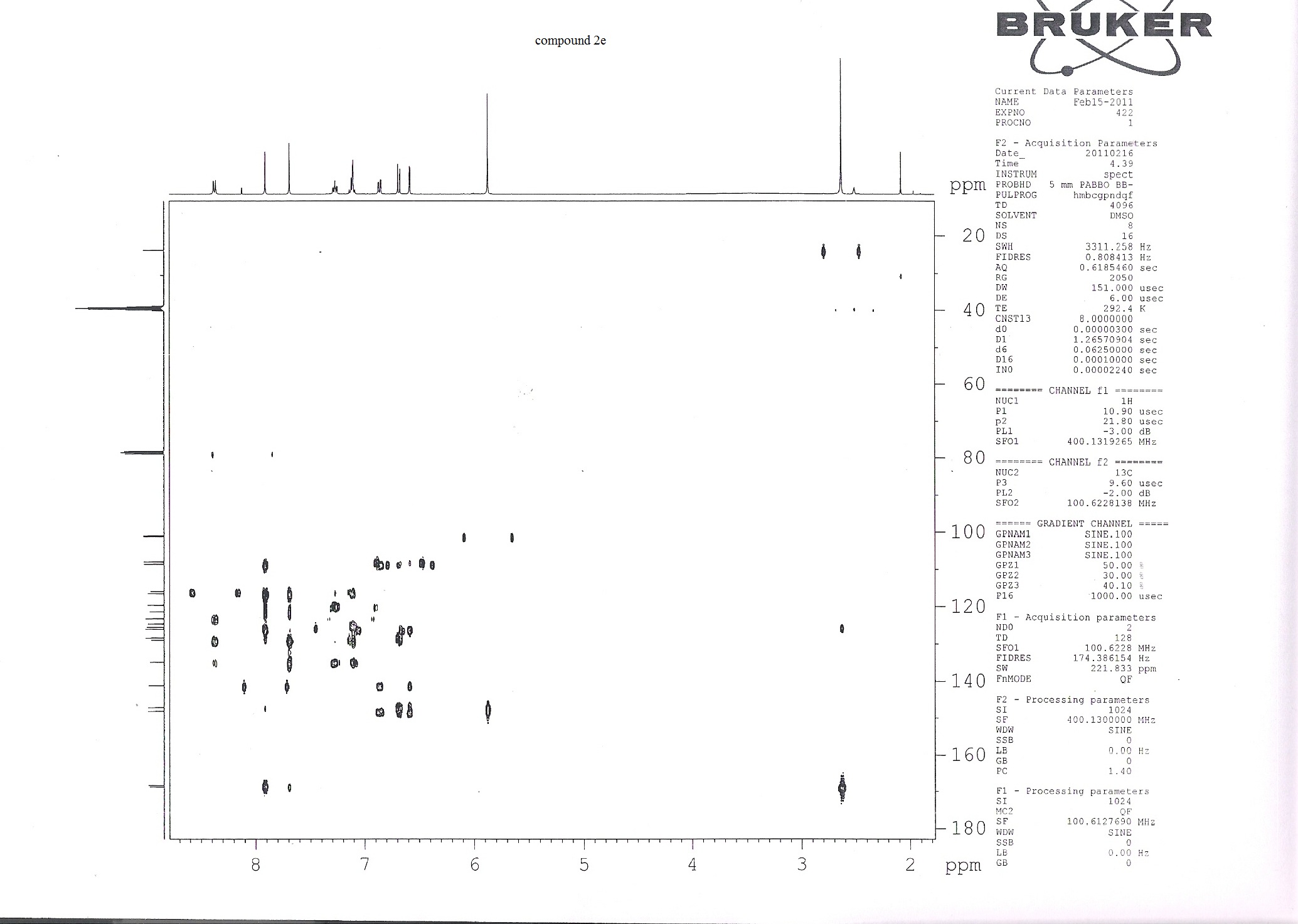

Supplement: Additional file 4 — Compound 2e. HMBC spectrum. [file 2191-2858-3-3-S4.jpeg]

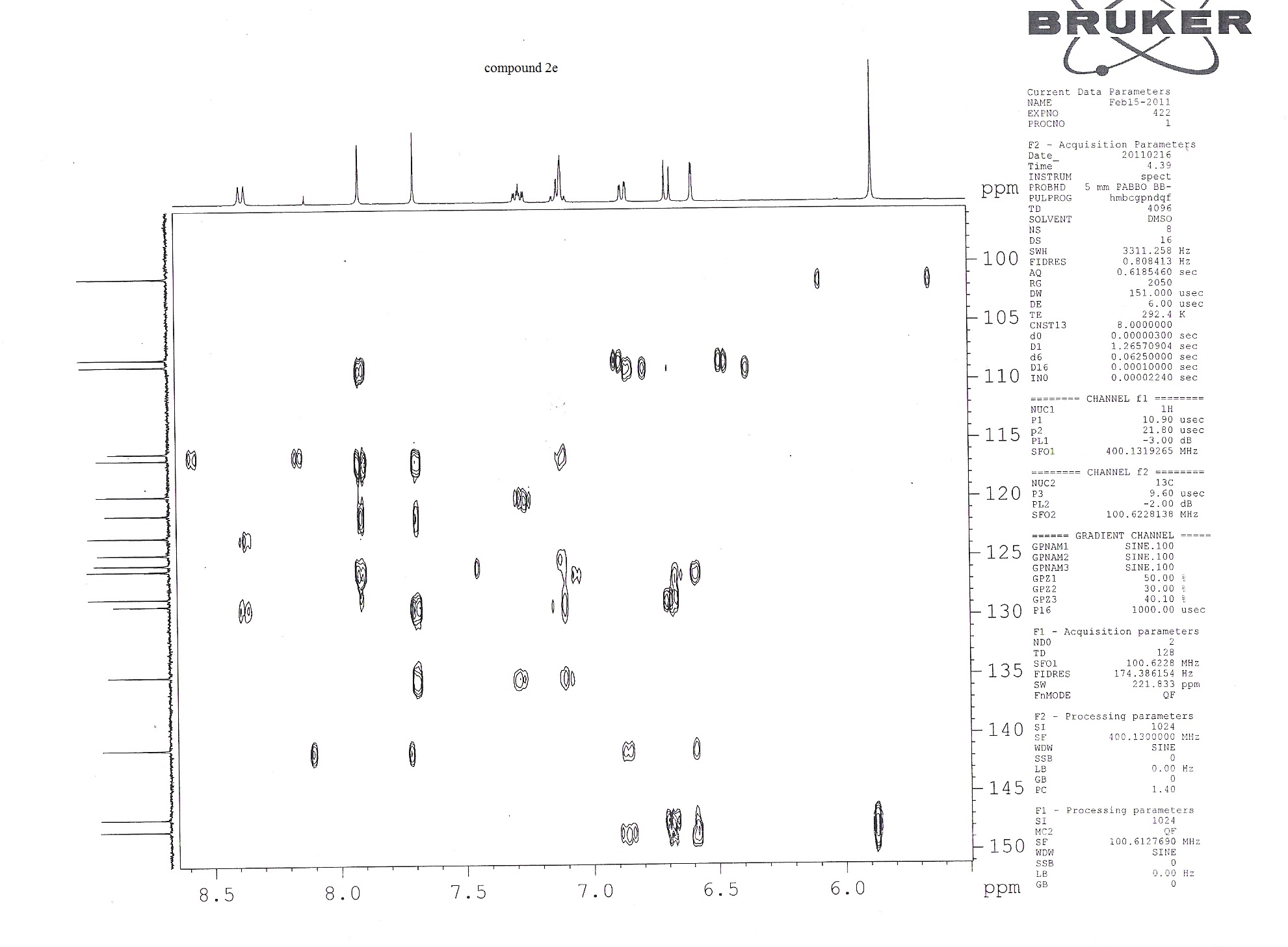

Supplement: Additional file 5 — Compound 2e. Expanded HMBC spectrum. [file 2191-2858-3-3-S5.jpeg]

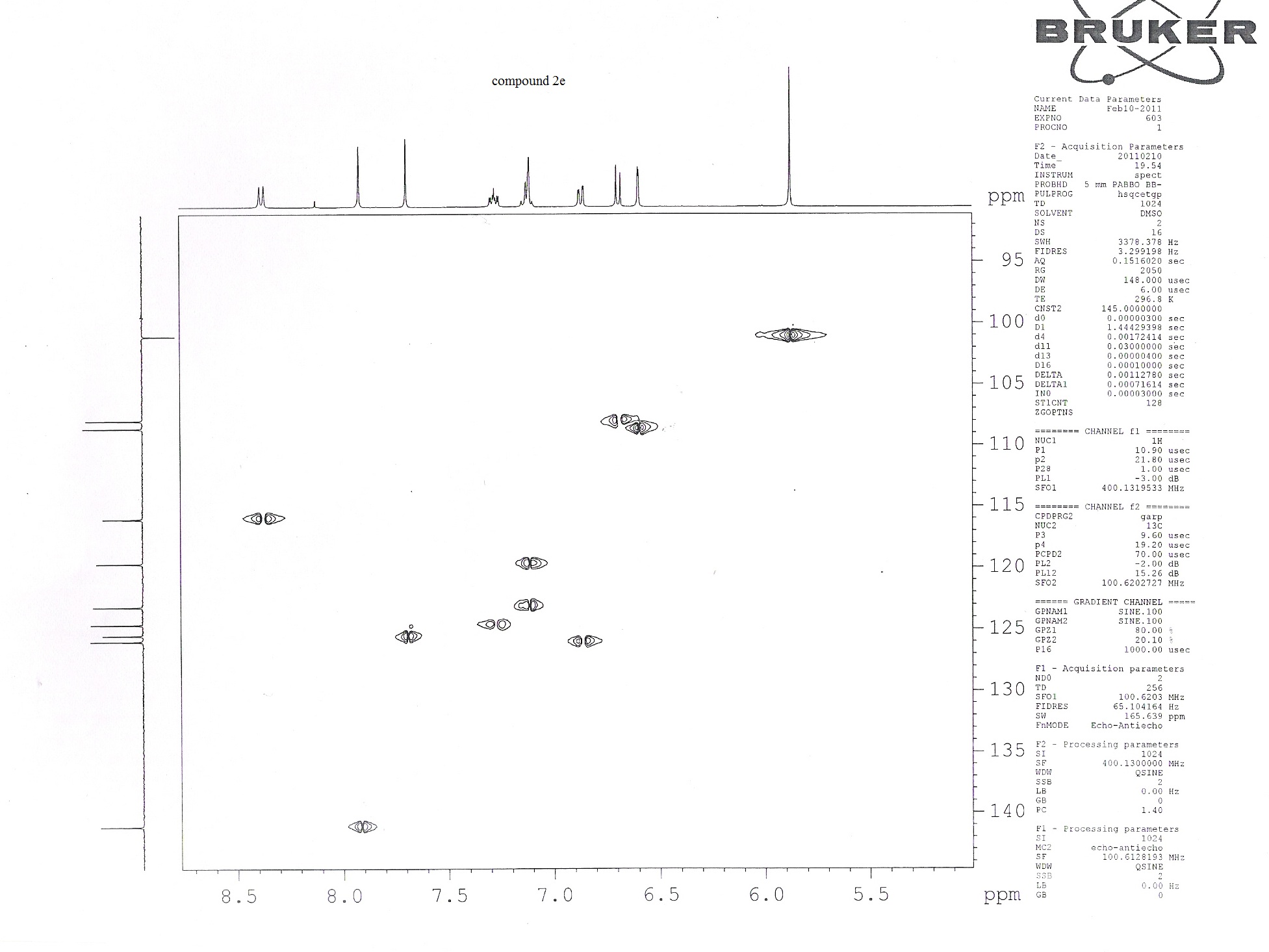

Supplement: Additional file 6 — Compound 2e. Expanded HSQC spectrum. [file 2191-2858-3-3-S6.jpeg]
